# Pathogenicity Islands in Uropathogenic *Escherichia coli* Clinical Isolate of the Globally Disseminated O25:H4-ST131 Pandemic Clonal Lineage: First Report from Egypt

**DOI:** 10.3390/antibiotics11111620

**Published:** 2022-11-13

**Authors:** Azza S. Zakaria, Eva A. Edward, Nelly M. Mohamed

**Affiliations:** Microbiology and Immunology Department, Faculty of Pharmacy, Alexandria University, Alexandria 25435, Egypt

**Keywords:** pathogenicity islands, uropathogenic *E. coli*, whole genome sequencing, ST131, virulence factors

## Abstract

Uropathogenic *Escherichia coli* (UPEC) is the main etiological agent of urinary tract infections (UTIs). The pathogenesis of UTIs relies upon UPEC’s acquisition of virulence determinants that are commonly inserted into large chromosomal blocks which are termed ‘pathogenicity islands’ (PAIs). In this study, we investigated the virulence-associated genes embedded in the chromosome of a UPEC Egyptian strain, EC14142. Additionally, we present a detailed characterization of the PAIs in the EGY_EC14142 chromosome. The isolate displayed a multidrug-resistant phenotype, and whole genome sequencing indicated that it belonged to the globally disseminated O25:H4-ST131 pandemic lineage and the *H*30-Rx clade. EGY_EC14142 carried genes that are responsible for resistance to aminoglycosides, fluoroquinolones, extended-spectrum β-lactams, macrolides, folate pathway antagonists, and tetracyclines. It encoded five PAIs with a high similarity to PAI II_536_, PAI IV_536_, PAI V_536_, PAI-536-icd, and PAI*usp*. The genome analysis of EGY_EC14142 with other closely related UPEC strains revealed that they have a high nucleotide sequence identity. The constructed maximum-likelihood phylogenetic tree showed the close clonality of EGY_EC14142 with the previously published ST131 UPEC international isolates, thus endorsing the broad geographical distribution of this clone. This is the first report characterizing PAIs in a UPEC Egyptian strain belonging to the globally disseminated pandemic clone O25:H4-ST131.

## 1. Introduction

Uropathogenic *Escherichia coli* (UPEC) is the main etiological agents of urinary tract infections (UTIs) including cystitis, pyelonephritis, and serious infectious complications that may lead to acute renal failure [1]. UPEC adopts a complicated pathogenic mechanism that is initiated through its efficient adherence to the superficial bladder epithelial cells, which is followed by the internalization of the bladder’s epithelium, replication, and the induction of host cytokine responses, thus establishing a UTI [2]. A core difference between UPEC and commensal *E. coli* is the expression of a wide array of virulence factors such as type 1 pili and adhesins, and both of these are necessary for the internalization step, P-fimbriae which enhances colonization and biofilm formation, toxins (cytotoxic necrotizing factor I and α-hemolysin), specific O antigens (O1, O2, O4, O6, O18), iron-uptake systems (yersiniabactin and aerobactin), capsules (K1, K5, K12), and factors mediating serum resistance [2,3,4]. This defined set of virulence determinants is commonly inserted into large chromosomal blocks which are termed ‘pathogenicity islands’ (PAIs) [3]. In the late 1980s, PAIs were first described in the UPEC strains 536 and J96 by Hacker et al. [5]. These clustered subsets of virulence attributes occupy relatively vast genomic regions ranging from 10 to 200 kb and reside in an area that is adjacent to the tRNA loci which act as anchor points, facilitating the insertion of foreign DNA through horizontal gene transfer [2]. Their G+C content and codon usage normally differ from that of the rest of the core genome [6]. In addition, PAIs carry various mobile genetic elements, including transposases, phage genes, integrases, and origins of replication. They are usually flanked by direct repeats (DRs), promoting the integration of bacteriophages and serving as recognition sites for enzymes that are responsible for the excision of PAIs from the bacterial chromosome, thus contributing to their genomic instability [2,6]. Seven PAIs have been identified in the prototype strain 536 which was isolated from a patient suffering from pyelonephritis. They had been designated as PAI I_536_ to PAI VII_536_ [4,7]. A sequence analysis of PAI I_536_ indicated the presence of an α-hemolysin gene cluster along with two genes showing homology to an F17-like and a CS12-like adhesin that exists in enterotoxigenic *E. coli* [3]. PAI II_536_ harbors a second α-hemolysin, the complete determinant for P-related fimbriae, and a gene cluster that is homologous with the filamentous hemagglutinin, *fha*, which is present in *Bordetella pertussis*. The open reading frame (ORF) in PAI II_536_ corresponding to *hra* is almost identical to that which is responsible for the heat-resistant agglutinin that is carried by *E. coli* O9:H10:K99 [3]. PAI III_536_ encodes a TSH-like hemoglobin protease, the *iro* siderophore system, S-fimbriae, a Sap adhesin, and an HmuR-like heme receptor [2]. PAI IV_536_ harbors a yersiniabactin siderophore system, the key element of the high pathogenicity island (HPI) of the pathogenic *Yersinia* spp. [2] which plays a chief role in the efficient colonization and fitness of *E. coli* [3]. The genetic structure of PAI V_536_ illustrated the presence of the K15 capsule determinant, and the Pix fimbriae determinant, together with genes that code for an autotransporter protein and a putative phosphoglycerate transport system [8]. Among the various proteins that are encoded by PAI VI_536_, eight proteins are non-ribosomal hybrid peptide synthetases/polyketide synthases (NRPS/PKS) [9]. PAI VII_536_ is a small island carrying genes of an undetermined function, except for two ORFs, one of which is associated with the biological switch for generating alternative type IV pili, and the other one codes for a histone-like protein [9]. Since the characterization of PAIs is an essential step to decipher the molecular mechanism of bacterial pathogenesis, we undertook this study to identify the PAIs in a UPEC Egyptian strain, EC14142. This is the first in silico analysis featuring the PAIs in a UPEC strain from Egypt.

## 2. Results and Discussion

### 2.1. Antimicrobial Susceptibility Profile

The antibiogram of EC14142, a strain that was isolated from the urine of a 72-year-old female patient admitted to Alexandria Main University Hospital (AMUH) in September 2019 with pyelonephritis, revealed a resistance to amoxicillin-clavulanate, cefepime, cefotaxime, ceftriaxone, ceftazidime, ciprofloxacin, colistin, levofloxacin, doxycycline, gentamicin, piperacillin-tazobactam, and sulfamethoxazole-trimethoprim (Table 1). The isolate possessed a multidrug-resistant phenotype, thus leaving the patient with limited treatment choices. A suspected previous exposure to third-generation cephalosporins, specifically, as continuous prophylaxis, in conjunction with comorbid illness requiring long hospitalization, are among the risk factors that are recognized for developing this resistance pattern [10]. The traditional first-line therapeutic choices for UTIs comprising cephalosporins, piperacillin-tazobactam, aminoglycosides, and sulfamethoxazole-trimethoprim [11] are ineffective against this uropathogen. Carbapenems seem to be the most appropriate treatment alternative in this case, however, their prudent use is required particularly in developing countries, such as Egypt, where antibiotics are available without prescription [12]. The susceptibility data are shown in Table 1.

### 2.2. Genomic Analysis of UPEC Strain EC14142

**Figure 1:**
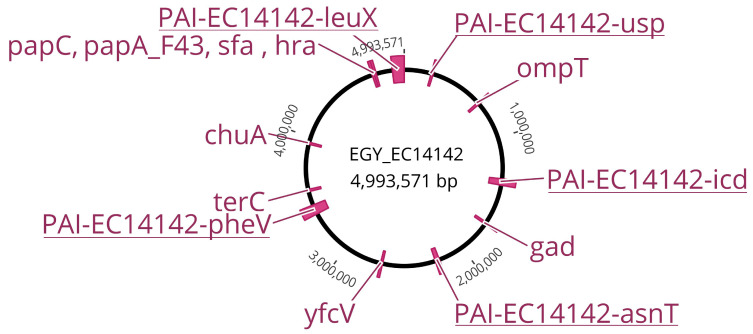
Comprehensive genetic map of the chromosome EGY_EC14142 belonging to the UPEC isolate EC14142. The map is based on the chromosome of *E. coli* K-12 MG1655 (NC_000913.3). PAIs and virulence factors encoding genes are indicated in accordance with their position on EGY_EC14142.

The whole genome sequencing of *E. coli* EC14142 was performed, and the de novo assembly resulted in 172 scaffolds, each with ≥500 bp. These scaffolds were used to construct the EGY_EC14142 chromosome of 4,993,571 bp with an overall G+C content of 51% and an N50 of 85375 (Figure 1). In addition, two plasmids, a Col(BS512) plasmid which was identified on a 2215 bp node, and an IncFIA/IncFII plasmid were identified in the genome of EC14142. The statistics of sequence assembly generated through the WGS are described in Supplementary Table S1. The genotyping of EC14142 revealed that the isolate belonged to the serogroup O25:H4, and the performed in silico sequence typing using the seven housekeeping genes scheme confirmed that the strain was of sequence type 131 (ST131) (Table 2).

The worldwide disseminated pandemic multi-resistant clone ST131 of the O25:H4 serotype is implicated as the major contributor to hospital- and community-acquired UTIs [13]. As published earlier, the demographic features and the clinical risk factors for colonization by the ST131 clone encompass a long hospitalization period [14], antibiotic exposure [15], being of the female sex [14], age [14,15], and the characteristics of the infection [16]. Isolated from a hospitalized 72-year-old female patient, the strain EC14142 typically complies with these risk factors. Although the data about the *E. coli* ST131 clone in Egypt are scarce, two studies indicated a high prevalence rate of this clone among UTI patients [12,17]. The gene coding for the uropathogenic specific protein (*usp*), a virulence factor mediating the pathogenesis of the *E. coli* urinary infection, was detected on the EGY_EC14142 chromosome, thus categorizing the EC14142 isolate as UPEC [18]. Previous studies have suggested that the *usp* gene is more likely to be observed in *E. coli* isolates of the ST131 clone [14,18]. Moreover, Clermont phylotyping assigned this isolate to phylogroup B2 by the allocation of the *chuA*, *fyuA*, and *yfcV* gene markers [19]. In general, *E. coli* belonging to the phylogenetic B2 group is recognized as the most dominating group of UPEC isolates worldwide [20]. The typing of the *fimH* gene (type 1 fimbrial adhesin is responsible for the adherence, invasion, and formation of intracellular bacterial communities [21]) demonstrated that the isolate carries a *fimH30* allele, resulting in clonotype CH40-30. Johnson et al. stated that the *fimH30* subtype is the most frequently identified among ST131 *E. coli* [22]. The *fimH30* subclonal lineage of ST131 (referred to as *H*30 or clade C in the Petty classification [23]) is known to express fluoroquinolone-resistant genes and some strains, which are designated as *H*30-Rx (clade C2), exhibit additionally the *bla*_CTX-M-15_ allele [24]. The resistome of EC14142 presented the two traits that were common to *H*30-Rx where the extended-spectrum β-lactam resistance was mediated through the *bla*_CTXM-15_ gene which is located on an IncFIA/IncFII plasmid and the fluoroquinolone resistance was mediated through the *aac(6′)-Ib-cr* gene which can be found on the above-mentioned plasmid as well as through mutations in chromosomally encoded genes: *parE* (I529L), *parC* (S80I, E84V), and *gyrA* (D87N, S83L). ResFinder identified an additional resistance to aminoglycosides (*aadA5*, *aac(3)*), sulphonamides (*sul1*), tetracycline (*tetA*), macrolides (*mphA*), β-lactams (*bla*_OXA-1_), and trimethoprim (*dfrA17*), which are all carried on the IncFIA/IncFII plasmid (Table 2).

### 2.3. Distribution of Virulence Genes on EGY_EC14142

The ST131-*H*30Rx clade was found to possess a greater fitness in comparison with other *E. coli*, allegedly controverting the long-established view that high levels of antibiotic resistance are compulsorily related to a fitness cost that minimizes the successful colonization of the host [25]. In accordance with this finding, besides its acquisition of various antibiotic resistance genes, EC14142 is heavily shaped by a variety of virulence factors-encoding genes that are located sporadically on its chromosome, EGY_EC14142 (Figure 1, Table 2). Among the autotransporters, EC14142 encoded the secreted autotransporter toxin, Sat, which is capable of conveying its secretion through the bacterial membrane [7]. Adhesins, which are known to increase the colonizing ability of an isolate via attachment to the uroepithelial cells [26], were demonstrated by the presence of *fimH*, *papA_F43*, *papC*, *iha*, *hra*, *yfcV* genes, and *sfa* operon. The restriction of iron availability, a traditional host defense mechanism against bacterial invasion, is successfully overpowered by *E. coli* ST131 through the secretion of various siderophores that bind to iron in the surrounding environment with a greater affinity than that of the host proteins [25]. The genes that are responsible for iron scavenging which are detected on the EGY_EC14142 chromosome included *chuA* and *iucC*. VirulenceFinder revealed some additional miscellaneous virulence genes such as the glutamate decarboxylase (*gad*) gene which enables the isolate to survive in the acidic urinary environment [27]. Serum resistance and evasion from complement-mediated damage are anticipated in the isolate EC14142 as it expresses the gene coding for serum resistance outer membrane lipoprotein (*traT*) and the outer membrane protein T (*ompT*), both of which are reported to promote the serum survival of the producing isolate [28]. EC14142 is expected as well to avoid tellurite toxicity and the consequent damage to the key cell components through the expression of the gene coding for tellurite ion resistance (*terC*) [29] (Figure 1, Table 2).

### 2.4. Structural Features of Pathogenicity Islands Integrated into EGY_EC14142 Chromosome

**Figure 2:**
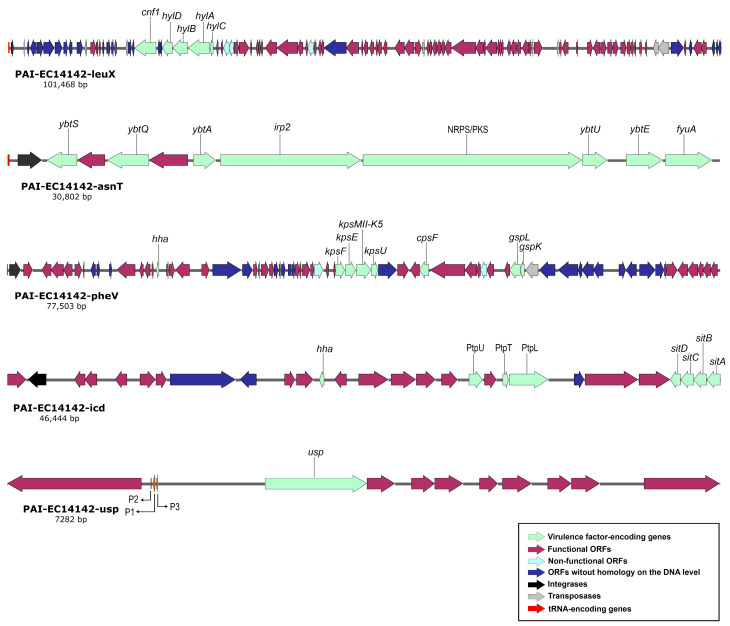
Schematic genetic representation of PAIs: PAI-EC14142-leuX, PAI-EC14142-asnT, PAI-EC14142-pheV, PAI-EC14142-icd, and PAI-EC14142-usp belonging to UPEC EC14142 isolate. The arrows indicate open reading frames (ORFs) with the green, purple, light blue, navy blue, black, grey, and red parts representing virulence factor-encoding genes, functional ORFs, non-functional ORFs (e.g., due to internal stop codons or frameshifts), ORFs without homology on the DNA level, integrases, transposases, and tRNA-encoding genes, respectively.

Generally, the pool of virulence-associated genes in UPEC isolates comprise genes that could be plasmid mediated, inserted sporadically on the chromosome, or embedded in huge uninterrupted regions of the chromosomal PAIs [5]. These are edged laterally by a tRNA, carry a gene for phage integrase, and their G+C content usually differs from the remaining part of the chromosome, revealing their “*en bloc*” horizontal acquisition [6]. With the data that were generated by the WGS, we were able to identify five pathogenicity islands which were integrated into EGY_EC14142 chromosome (Table 3). PAI-EC14142-leuX (GenBank accession number: OM100123) was found to be about 101 kb in size, and it carries genes encoding two toxins; the α-hemolysin (*hlyCABD*) operon which promotes the passage of bacterial cells to blood, and the classical phylogroup B2 cytotoxic necrotizing factor (*cnf1*) which has been stated to cause the necrosis of the host endothelial cells [3,26]. This PAI had a G+C content of 46.3%. It was inserted next to *leuX* tRNA loci, and it showed 99.7% of the nucleotide identity (66% of the sequence length) to PAI II_536_ belonging to the prototype UPEC strain 536. Within a PAI, the bacteriophage’s integrases play an important role in catalyzing the recombination between the attachment sites *attP* and *attB* that are present on the bacteriophage and at the 3′ end of bacterial tRNA, respectively, facilitating the horizontal transfer of the PAI [30]. The in silico analysis of the genetic structure of PAI-EC14142-leuX confirmed the presence of an integrase with similarity to the phage P4 integrase C-terminal catalytic domain (INT_P4_C) immediately downstream of *leuX* tRNA segment (Figure 2). As a general rule, PAIs tend to be unstable and capable of spontaneous deletion, a prerequisite step to horizontal transfer [7]. Consequently, the appearance of non-hemolytic colonies was described in isolates experiencing the deletion of PAI I_536_ or PAI II_536_ from their chromosomes due to the loss of the *hlyCABD* operon. Additionally, a study analyzing the virulence potential of a UPEC strain in vivo reported a decrease from 2 to 3 logs in LD_50_ coinciding with the loss of PAI II_536_ [9]. The excision of PAI II_536_ is facilitated by the site-specific recombination of DRs, a repeated sequence of 18 bp, flanking the island and resembling the prophage’s left and right ends (*attL* and *attR*) [30]. We were able to detect a complete 18-bp sequence on the left border of PAI-EC14142-leuX and a truncated sequence on its right border, signifying an impossible homologous recombination and reflecting the partial stability of this island. A sporadic arrangement of transposase genes and insertion sequence (IS) elements was noticed throughout PAI-EC14142 leuX, which encoded six putative transposases and five IS elements (IS*2*, IS*3*, IS*21-like*, IS*629*, and IS*1341*), creating a mosaic-like organization of the island (Figure 2).

PAI-EC14142-asnT (GenBank accession number: **OM100124**) was about 31 kb in size, and it was present in the vicinity of *asnT* tRNA. It had a G+C content of 58.2%, and it was nearly identical (99.5% of the nucleotide identity; 99.9% of the sequence length) to PAI IV_536_. A bacteriophage P4-like integrase (*intB*) was located downstream of the tRNA gene *asnT*. PAI IV_536_ was originally discovered in *Yersinia pestis*, and it is also called HPI. It encodes the yersiniabactin sequestering system (Ybt) that is essential for the fitness of the UPEC isolates and the membrane transporter *fyuA,* which participates as well in the biosynthesis of yersiniabactin siderophore [7]. PAI-EC14142-asnT carries both of these virulence determinants together with the *irp2* gene encoding the iron-repressible high-molecular-weight protein (Irp2) that is involved in the production of yersiniabactin. A recent observation implicates a multifunctional metallophore role of the Ybt system, where besides its iron-sequestering activity, it binds to Cu^2+^ and decreases its toxicity in the bacterial cell. Meanwhile, in low-copper environments, the Ybt system imports this biometal which is essential for the enzymatic function of *E. coli* amine oxidases, implementing a strategy that is denoted as “nutritional passivation” [31]. The multi-modular enzyme system of non-ribosomal peptide synthetases/polyketide synthases (NRPS/PKS) secretes the genotoxin colibactin, a virulent and suspected procarcinogen contributing to bacterial fitness during extraintestinal infections [7]. The NRPS/PKS assembly was located on PAI-EC14142-asnT. Although previous studies referred to the NRPS/PKS assembly as an integrative part of PAI VI_536_ [7,9], we encountered this multi-enzymatic module embedded into PAI-EC14142-asnT, implying a prior existence of an island with a similarity to PAI VI_536_ in this chromosomal position that was possibly deleted, leaving behind the NRPS/PKS assembly. Comparable to PAI IV_536_, the flanking repeat structures were absent from PAI-EC14142-asnT (Figure 2).

The island PAI-EC14142-pheV (GenBank accession number: OM100125) was integrated at the *pheV* tRNA segment, had a size of 77.5 kb, and encoded the phage integrase P4 integrase C-terminal catalytic domain (INT_P4_C) immediately downstream of *pheV* (Table 3). The island showed a similarity to PAI V_536_ (98.6% of the nucleotide identity; 44% of the sequence length). The G+C content of PAI-EC14142-pheV reached 47.9%. The island’s rich content of A+T could be evidence of its recent acquisition since over time, the G+C content increases, and the sequence composition of the island becomes comparable to that of the core genome [7]. The secretion of capsular polysaccharide (CPS or *K*-antigen) allocates a negative charge on the bacterial surfaces, thus rendering the bacteria hydrophilic and shielded from mucus entrapment [32]. In addition, these exopolysaccharides block the activation of the complement pathway in the host, thus providing the producing bacteria additional protection from phagocytosis and serum killing processes [33]. PAI-EC14142-pheV harbored the K5 variant of the group II capsule (*kpsMII-K5*), along with the capsular polysaccharide biosynthesis and the export inner-membrane genes, *cpsF* and *kpsE*, respectively (Table 3). The inner membrane platform (*gspL*) and minor pseudopilin (*gspK*) genes detected in PAI-EC14142-pheV belong to the general secretion pathway, GSP, and are reported to be involved in the translocation of proteins from the bacterial periplasm across the outer membrane and into the extracellular surroundings [34]. The Hha protein located on PAI-EC14142-pheV is known to modulate the expression of several virulence genes, among which is the *hlyCABD* operon encoding the α-hemolysin toxin. This modulation seems to be tightly associated with the environmental parameters such as osmolarity and temperature as previously published [35].

PAI-EC14142-icd (GenBank accession number: OM100126) had an approximate size of 46 kb, a G+C content of 51.1%, and it was associated with *icd* chromosomal insertion site. It possessed a Φ21-type-specific phage integrase directly downstream of *icd* (Figure 2). It shared a high degree of similarity (96.9% of the nucleotide identity; 77% of the sequence length) with PAI-536-icd. This island carried genes encoding the *sitABCD* operon, an ATP-Binding Cassette (ABC) transporter of iron/manganese in the UPEC isolates. The transition metals, iron and manganese, serve as cofactors for the metalloprotein enzymes in bacterial pathogens which depend on the presence of these sophisticated transport systems to gain access to free or chelated metals for the colonization of their hosts [36]. The gene *iss*, which was detected on PAI-EC14142-icd, encodes the enhanced serum survival (Iss) protein which is predicted to inhibit the activation of the complement cascade and the formation of the membrane attack complex (MAC) in the serum of the host. In promoting immune evasion, *iss* restrains MAC from its ability to form pores in the bacterial outer membrane, thus improving the survival level of the pathogen in serum [36]. Furthermore, PAI-EC14142-icd encoded the phage tail genetic elements *ptpT*, *ptpU*, and *ptpL*, providing additional intracellular survival level and virulence to the producing isolate (Table 3) [37].

The fifth island which was detected on the EGY_EC14142 chromosome, PAI-EC14142-usp (GenBank accession number: OM100127), is a small island of an approximate size of 7 kb, a G+C content of 44%, and it harbors just the uropathogenic specific protein-coding gene, *usp*, with three closely associated *imu1-3* genes (Figure 2). It was highly similar (97.9% of the nucleotide identity; 99% of the sequence length) to PAI*usp*, thus belonging to UPEC strain UTI89 (GenBank accession number: NC_007946). This island is inserted into the *aroP*-*pdhR* intergenic region and is present in all of the *usp*-positive *E. coli* genome sequences that are available on the GenBank database. The Usp is a genotoxin that has been shown to be active against mammalian cells and to provoke pyelonephritis and bacteremia in patients who are infected with the UPEC strain producing this toxin [38]. The three *imu1-3* genes located downstream of *usp* are essential for protecting the producer strain against its own toxin. The flanking genes of PAI*usp*, *aroP* and *pdhR* correspond to the membrane protein that imports aromatic amino acids into the bacterial cell and the pyruvate dehydrogenase complex regulator which controls respiration in *E. coli*, respectively. The expression of *usp* is reported to be regulated by the P1 promotor, which is present in the upstream region of *aroP*, and it was experimentally proven to be induced by environmental factors such as the concentration of aromatic amino acids, temperature, and urea accumulation [38].

### 2.5. Genomic Comparisons of EGY_EC14142 with Closely Related UPEC Strains

**Figure 3:**
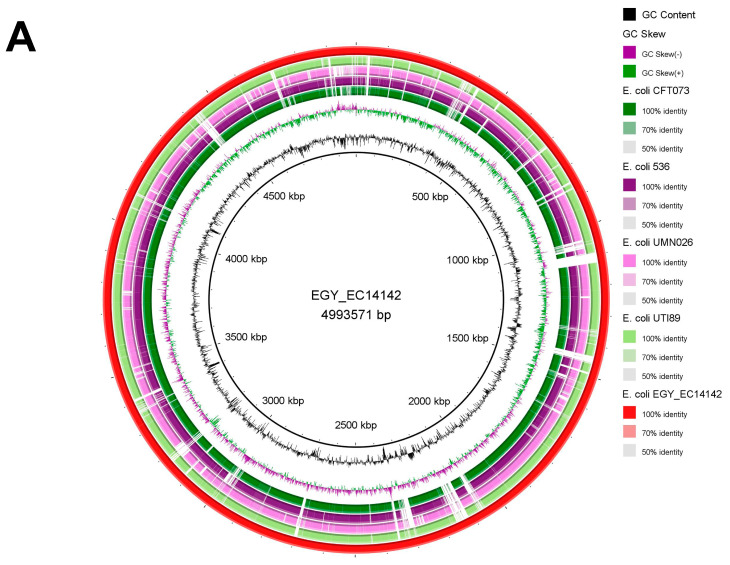

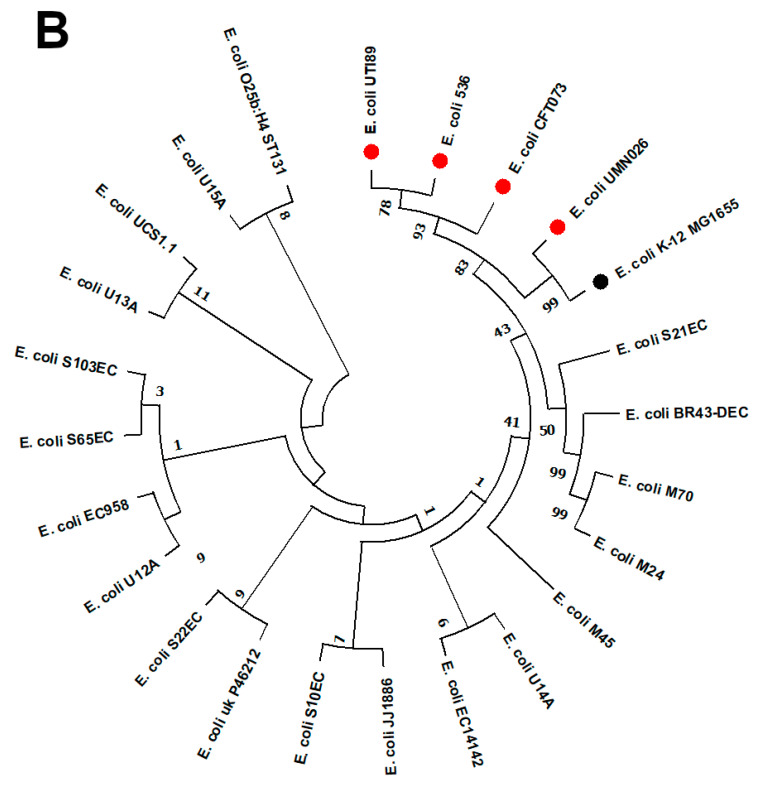
(**A**) Circular genome comparison of EGY_EC14142 chromosome with other chromosomes of UPEC strains. Each of the genome sequence assemblies of UPEC strains was aligned against EGY_EC14142 using the Basic Local Alignment Search Tool nucleotide tool (BLASTn) and visualized using the BLAST Ring Image Generator (BRIG) tool. The outermost ring (red) corresponds to EGY_EC14142 chromosome belonging to EC14142 strain, with its size being displayed in the middle of the ring. Next, UTI89 (NC_007946) is shown (light green). The third ring corresponds to UMNO26 (NC_011751) (pink), then, 536 (NC_008253) (purple) and CFT073 (NC_004431) (dark green) are shown. Genomic regions covered by BLASTn are represented by a solid color in concentric rings (with varying color degrees depending on percentage identity), whereas white gaps indicate genomic regions not covered by BLASTn. (**B**) Phylogenetic tree of EC14142 relative to 22 UPEC strains. The phylogenetic tree was generated using MEGA software (v11.0.9) with the maximum likelihood approach according to the aligned sequences by Clustal Omega. *E. coli* K-12 MG1655 (NC_000913.3) was set as a reference, and it is displayed in black. Referral strains, 536 (NC_008253), CFT073 (NC_004431), UTI89 (NC_007946), and UMNO26 (NC_011751) are indicated by red bullets. A bootstrap replication value of 100 was applied.

The sequence of the EGY_EC14142 chromosome was compared to the chromosomes of the UPEC strains which are available on the GenBank database where each of the genome sequence assemblies of these strains were aligned against EGY_EC14142 using the BLASTn tool, then, they were visualized using the BRIG tool (Figure 3A). The BLASTn comparison to CFT073 (NC_004431) and 536 (NC_008253) showed a high nucleotide identity of 99.1% with 86% and 88% sequence lengths, respectively. The comparative chromosomal analysis of EGY_EC14142 with UTI89 (NC_007946) revealed a nucleotide identity of 98.9% (88% sequence length). A similar genetic organization was depicted upon comparing the chromosomes of EC14142 and UMN026 (NC_011751) with a percentage identity of 97.8% (80% sequence length). Despite the detected similarity between EGY_EC14142 and the chromosomes of the UPEC strains. The BLAST comparison was followed by BRIG visualization revealed the unique genomic sequence of EGY_EC14142 which was evidenced by the white gaps appearing through the concentric rings generated by BRIG or the difference in the percentages of the sequence length as calculated by BLASTn (Figure 3A).

### 2.6. Phylogenomic Analysis of EC14142 Related to E. coli Belonging to ST131 Clade C

A maximum likelihood phylogenetic tree of 23 UPEC strains (19 UPEC belonging to ST131 clade C, including EC14142, and four referral strains) was constructed using MEGA software (v11.0.9) with 100 bootstraps (Figure 3B). *E. coli* K-12 MG1655 was set as a reference. This clone has become the most predominant lineage that is associated with a variety of infections around the globe [21,39]. Moreover, the ST131 clone is successfully disseminated worldwide due to its virulence and multidrug resistance ability as well as its epidemic potential [13,39]. Upon inspecting the generated phylogenetic tree, it was noticed that, although they were residing on distinct branches, the compared isolates showed relatedness. EC14142 was clustered with *E. coli* U14A (CP035516.1) which was recovered from a urine sample in Australia. This cluster exhibited relatedness to *E. coli* M45 (CP080119.1), *E. coli* M24 (CP080120.1), and *E. coli* M70 (CP080118.1) which were obtained from different urine samples in the Czech Republic, while *E. coli* BR43-DEC (CP035377.1) was obtained from a uropathogenic clinical sample in Brazil, and *E. coli* S21EC (CP076689.1) was isolated from a UTI patient in the United Kingdom. The referral strains were subclustered from a single branch, indicating the close clonality of these strains.

We understand and acknowledge the limitations of this study. The characterization of the PAIs in the UPEC isolate EC14142 was based on the in silico analysis which may limit the association of the genomic features of these PAIs to the virulence characteristics of the isolate. The phenotypic determination of the virulence factors could have enabled the correlation of the virulence-associated genes that are embedded in the PAIs to the virulence potential of the isolate.

## 3. Materials and Methods

### 3.1. E. coli EC14142 Strain Isolation and Identification

A clinical *E. coli* strain (EC14142) was obtained from the microbiology laboratory facility of Alexandria Main University Hospital (AMUH), which is the main referral hospital in the northern sector of Egypt, with approximately 100,000 total hospital admissions per year. The strain was isolated from the urine of a 72-year-old female patient admitted to AMUH in September 2019 with pyelonephritis. The collected sample was preserved in a Luria-Bertani broth (LB, HiMedia Lab., Mumbai, India) supplemented with 15% glycerol, and it was kept at −80 °C. For the identification, it was plated onto MacConkey and eosin methylene blue (Oxoid, Hampshire, UK) agar plates. Following the incubation at 37 °C for 24 h, the colonies from the pure cultures were identified by Gram staining and standard biochemical tests including triple-sugar iron, citrate utilization, and urease tests. The sample was characterized using a Vitek^®^ 2 Advanced Expert System™ (BioMèrieux, La-Balme-les-Grottes, France).

### 3.2. Antimicrobial Susceptibility Testing

The antimicrobial susceptibility testing was performed using the disc diffusion method (Kirby-Bauer) on Mueller–Hinton agar (Difco-BBL, Detroit, MI, USA) according to Clinical Laboratory Standards Institute (CLSI, 2020) [40]. Fourteen antimicrobial discs (Oxoid, Hampshire, UK) were used including amoxicillin-clavulanate, cefepime, cefotaxime, ceftriaxone, ceftazidime, ciprofloxacin, colistin, doxycycline, imipenem, gentamicin, levofloxacin, meropenem, piperacillin-tazobactam, and sulfamethoxazole-trimethoprim. The results were interpreted according to CLSI [40], except for colistin since the disk diffusion test, which is commonly used in clinical laboratories, is unreliable because colistin diffuses poorly into agar due to it performing electrostatic interactions with the acid or sulfate groups of the agar, resulting in smaller inhibition zones and high error rates when it is compared to the broth microdilution method [41]. Minimum inhibitory concentrations (MICs) of the above-mentioned antibiotics were determined by the broth microdilution technique in triplicates using the Muller–Hinton broth (Difco-BBL, Detroit, MI, USA). The reference strain, *E. coli* ATCC 25922, was used as a quality control strain.

### 3.3. DNA Extraction and Whole Genome Sequencing (WGS)

The *E. coli* strain EC14142 was cultivated on sheep blood agar at 37 °C overnight prior to DNA isolation by Invitrogen Easy-DNATM kit (Invitrogen, San Diego, CA, USA). The DNA concentration was quantified on a Qubit^TM^ 2.0 fluorometer using the dsDNA BR assay kit (Invitrogen, San Diego, CA, USA). The genomic DNA was prepared for the Illumina pair-end sequencing as per the Illumina NexteraXT^®^ DNA Library Prep Guide Document # 15031942 v05 May 2019 by following the protocol. The library was sequenced using the Illumina MiSeq using MiSeq reagent kit v2 and 500 cycles with a standard flow cell. The sequencing yield per sample ranged from 650 Mb to 1615 Mb, totaling 19,535 Mb across all of the samples. The 2 × 120 bp paired-end Illumina reads files passed the standard quality checks according to the software package FastQC v 0.11.7 (Babraham Bioinformatics, Cambridge, UK). The reads were then trimmed and de novo assembled by the SPAdes software (v3.15.3) (https://cab.spbu.ru/software/spades/, accessed on 25 March 2022) using the default settings. To build the assembly graphs, a low *k*-mer (*k* = 31) and a high *k*-mer (*k* = 127) were applied as low *k*-mers allow for the discovery of variants at relatively lower coverage, while genome complexity and large structural variations are more accessible at high *k*-mers [42]. The assemblies were filtered, and nodes of more than 500 bp were retained, and the obtained scaffolds were analyzed to confirm the species and serotype of *E. coli* strain utilizing the pipelines of the Center for Genomic Epidemiology (CGE) (https://cge.cbs.dtu.dk/services/, accessed on 30 March 2022) using the default settings. The plasmids were identified using PlasmidFinder v.2.1 (https://cge.food.dtu.dk/services/PlasmidFinder, accessed on 30 March 2022). The O- and H-types were identified by SeroTypeFinder v 2.0 (https://cge.cbs.dtu.dk/services/SerotypeFinder/, accessed on 30 March 2022). The profiles of the virulence and antimicrobial resistance were determined by VirulenceFinder v2.0 (https://cge.cbs.dtu.dk/services/VirulenceFinder/, accessed on 30 March 2022) and ResFinder v4.1 (https://cge.cbs.dtu.dk/services/ResFinder/, accessed on 30 March 2022), respectively. The clonotype of the isolate was determined by CHTyper v1.0 (https://cge.cbs.dtu.dk/services/CHTyper/, accessed on 30 March 2022). The sequence type (ST) of the isolate was determined based on the seven housekeeping genes (*adk*, *fumC*, *gyrB*, *icd*, *mdh*, *purA*, and *recA*) scheme that are available on CGE (MLST, v2.0, https://cge.cbs.dtu.dk/services/MLST/, accessed on 30 March 2022).

### 3.4. Construction of EGY_EC14142 Chromosome and Identification of Its PAIs

The generated contigs with hits of *>* 98% identity were combined manually and aligned against the prototype UPEC strain 536 to be used as a reference sequence using Basic Local Alignment Search Tool nucleotide (BLASTn) (https://blast.ncbi.nlm.nih.gov/Blast.cgi, accessed on 5 April 2022) to obtain the whole chromosome sequence designated as EGY_EC14142. The constructed chromosome was mapped against *E. coli* (taxid:562) using BLASTn and annotated using NCBI Prokaryotic Genome Annotation Pipeline (PGAP) (NCBI Prokaryotic Genome Annotation Process (nih.gov)). The map of EGY_EC14142 was displayed through Geneious software (v2022.0, Biomatters, https://www.geneious.com, accessed on 5 April 2022).

The specific ORFs and virulence markers of PAI-EC14142-leuX and PAI-EC14142- PheV were analyzed by a BLASTp-based bidirectional best hit with a minimal coverage of 80% and an e-value cut-off of 1 × 10^−4^ using the amino acid sequences of PAI II_536_ and PAI V_536_ (GenBank accession numbers: AJ494981 and AJ494981), respectively. PAI-EC14142-asnT, PAI-EC14142-icd, and PAI-EC14142-usp were identified through alignment with a minimal coverage of 80% against the complete chromosomes of UPEC strains 536 and UTI89 which were deposited in GenBank under the accession numbers NC_008253 and NC_007946, respectively. The insertion sequence elements of the different PAIs were identified using MobileElementFinder (v1.02, https://cge.cbs.dtu.dk/services/MobileElementFinder/, accessed on 9 April 2022) which are available on CGE. The islands were annotated, and their genetic structures were schematically presented utilizing the SnapGene software (v5.2, Insightful Science, www.snapgene.com, accessed on 9 April 2022).

### 3.5. Genomic Similarity and Phylogenetic Analysis

The BLAST Ring Image Generator (BRIG) tool (http://sourceforge.net/projects/brig, accessed on 20 April 2022) was used to create a circular schematic map to compare the chromosome EGY_EC14142 to the chromosomes of other UPEC strains, namely, 536 (NC_008253), CFT073 (NC_004431), UTI89 (NC_007946), and UMN026 (NC_011751). In addition, Clustal Omega, a multiple sequence alignment program that is available through the Geneious software (v2022.0, Biomatters, https://www.geneious.com, accessed on 9 April 2022), was used to align the genome of EC14142 with the genome sequences of 22 UPEC strains that are available on the database as well as with the genome of *E. coli* K-12 MG1655 (NC_000913.3). Following the alignment, a phylogenetic tree was constructed with the maximum likelihood phylogeny, a GTR model of the nucleotide substitution, a GAMMA distribution of the rate heterogeneity, and 100 bootstrap replicates by applying the MEGA software v11.0.9 (https://megasoftware.net/, accessed on 20 April 2022). The comparison included 19 isolates belonging to ST131 clade C and the referral strains 536, CFT073, UTI89, and UMN026. The closed genome of *E. coli* K-12 MG1655 was set as a reference.

## 4. Conclusions

In conclusion, we provide here the first in silico detailed characterization of PAIs in a UPEC strain of the O25:H4-ST131 pandemic lineage isolated from a UTI patient admitted to a tertiary hospital in Alexandria, Egypt. The MDR phenotype and the rapid global dissemination highlight the crucial necessity to better understand this high-risk clone and to tackle its escalation, especially in a low-income country that is struggling with a high burden of infectious diseases such as Egypt. The problem is aggravated when this lineage acquires large genomic regions encoding for a wide array of virulence factors, known as PAIs, which are capable of spontaneous excision and integration via horizontal transfer, thus resulting in a continuous evolution of the pathogen. The close relatedness of EC14142 from Egypt, encoding five PAIs in its chromosome, with previously published PAI-bearing UPEC international isolates endorses the broad geographical distribution of this clone. The identification of PAIs in UPEC strains is a fundamental step towards the selection of appropriate treatment options and the accurate differentiation of UPEC pathotypes, hence, this aids epidemiology.

## Figures and Tables

**Table 1:** Antimicrobial susceptibility profile of *E. coli* strain EC14142 from Egypt.

Antibiotics/Disc Code	Disc Content, µg/Sensitivity Pattern	MIC, µg/mL/ Sensitivity Pattern
Amoxicillin-clavulanate/AMC	30/R	64/R
Cefepime/FEB	30/R	1024/R
Cefotaxime/CTX	30/R	1024/R
Ceftazidime/CAZ	30/R	1024/R
Ceftriaxone/CRO	30/R	≥4096/R
Colistin/CT	25/I	4/R
Ciprofloxacin/CIP	5/R	256/R
Doxycycline/DO	30/R	16/R
Imipenem/IPM	10/S	1/S
Gentamicin/CN	10/I	1/R
Levofloxacin/LEV	5/R	16/R
Meropenem/MEM	10/S	0.25/S
Piperacillin-tazobactam/TZP	110/R	64/R
Sulfamethoxazole-trimethoprim/SXT	25/R	16/R

R indicates resistance; S indicates sensitive isolate; I indicates intermediately resistant isolate.

**Table 2:** Molecular typing, resistance profile, and virulence genes encoded by the genome of UPEC isolate EC14142 from Egypt.

Serotype ^a^	Phylogroup ^b^	Pathotype ^c^	ST ^d^	Clonotype ^e^	Virulence Profile ^f^		Resistance Profile ^g^	
					Virulence Factor	Virulence Gene	Antimicrobial Class	Antimicrobial Resistance Genes
O25:H4	Group B2	UPEC	131	CH40-30	Heme binding outer membrane	*chuA*	β-lactams	*bla*_OXA-1_, *bla*_CTX-M-15_
					D-mannose-specific adhesin, type 1 fimbriae	*fimH*	Folate pathway antagonist	*sul1*, *dfrA17*
					Glutamate decarboxylase	*gad*	Tetracyclines	*tet(A)*
					Heat-resistant agglutinin	*hra*	Fluoroquinolones	*aac(6’)-Ib-cr*
					Adhesin siderophore receptor	*iha*	Aminoglycosides	*aadA5*, *aac(3)*
					Ferric aerobactin synthetase	*iucC*	Macrolides	*mph(A)*
					Outer membrane protein (protease) T	*ompT*		
					Pilin subunit F43. P Fimbriae usher	*papA_F43*		
					P fimbriae operon. Pilus assembly	*papC*		
					Secreted autotransporter toxin	*sat*		
					S-fimbrial adhesin	*sfa*		
					Tellurite resistance protein	*terC*		
					Outer membrane lipoprotein	*traT*		
					Putative chaperone-usher fimbria	*yfcV*		

^a^ Data obtained from SeroTypeFinder version 2.0; ^b^ Based on ClermonTyping method; ^c^ Pathotype: based on the presence of *chuA*, *fyuA*, vat, and *yfcV* virulence genes; ^d^ ST: sequence type, data obtained from MLSTFinder version 2.0; ^e^ Clonotype: determined according to *fumC*- *fimH* alleles, data obtained from CHTyper version 1.0; ^f^ Virulence profile: virulence genes as obtained from VirulenceFinder version 2.0; ^g^ Antimicrobial resistance profile as obtained from ResFinder version 4.0.

**Table 3:** Features of pathogenicity islands carried on EGY_EC14142 chromosome of UPEC isolate EC14142 from Egypt.

PAIs (GenBank Accession No.)	Size (bp)	G+C Content (%)	Position in Chromosome	No. of ORFs	Main Virulence Determinants Encoded within the PAI		Ref.
					Gene/Protein (Virulence Factor)	Function or Homology	
PAI-EC14142-leuX (OM100123)	101,468	46.3	4646–4,896,778	99	*cnf1* (cytotoxic necrotizing factor 1)	Engaged in cell necrosis	[26]
					*hylCABD* (α-hemolysin)	Promotes passage of bacteria into the blood	[3]
PAI-EC14142-asnT (OM100124)	30,802	58.2	2,211,273–2,242,074	25	*fyuA* (ferric yersiniabactin uptake A)	Acts as an outer-membrane receptor for iron uptake	[25]
					*irp2* (iron-repressible gene)	Involved in the biosynthesis of yersiniabactin siderophore	[2,3]
					*ybtA*, *ybtE*, *ybtQ*, *ybtS*, *ybtU* (yersiniabactin iron-capture island)	Involved in biosynthesis, regulation, and transfer of yersiniabactin siderophore	[2,3]
					NRPS/PKS (non-ribosomal peptide synthetase/polyketide synthase system)	Causes the breakage of DNA and cell cycle arrest in human cells	[9]
PAI-EC14142-pheV (OM100125)	77,503	47.9	3,357,439–3,434,941	79	*kpsE*, *kpsF*, *kpsU* (capsule polysaccharide export inner-membrane system)	Promotes polysaccharide translocation across the inner membrane to the cell surface	[33]
					*kpsMII-K5* (K5 variant of group II capsule)	Involved in capsular polysaccharide production	[33]
					*cpsF* (capsular polysaccharide biosynthesis gene)	Prevents the activation of host phagocytic activity	[32]
					*gspL*, *gspK* (general secretion pathway)	Exports proteins from the bacterial cytoplasm	[34]
					*hha* (high hemolytic activity modulator)	Modulates expression of bacterial virulence factors	
PAI-EC14142-icd (OM100126)	46,444	51.1	1,340,973–1,387,416	61	*iss* (increased serum survival)	Involved in serum resistance and evasion from phagocytosis	[28]
					PtpT, PtpU, PtpL (phage tail proteins)	Increase resistance and virulence	[37]
					SitDCBA (iron/manganese transport system)	Involved in the transportation of Fe^2+^ and Mn^2+^ ions	[36]
PAI-EC14142-usp (OM100127)	7282	44	228,116–235,397	10	*usp* (uropathogenic-specific gene)	Associated with pyelonephritis and bacteremia in an infected host	[38]

## Data Availability

The EC14142 whole genome shotgun sequence was deposited in DDBJ/ENA/GenBank under the Bioproject accession number (http://www.ncbi.nlm.nih.gov/bioproject/784618, accessed on 29 April 2022) and reference BioSample accession **SAMN23497059** (https://www.ncbi.nlm.nih.gov/biosample/23497059, accessed on 29 April 2022). The raw sequence data have been submitted to the Sequence Read Archive (SRA) (https://submit.ncbi.nlm.nih.gov/about/sra/, accessed on 9 April 2022) under study accession number **PRJNA784618** (https://www.ncbi.nlm.nih.gov/sra/PRJNA784618, accessed on 9 April 2022). The de novo assembly of the five PAIs detected in the current study were deposited in NCBI using BankIt tool under the accession numbers (OM100123-OM1OO127) for PAI-EC14142-leuX, PAI-EC14142-asnT, PAI-EC14142-pheV, PAI-EC14142-icd, and PAI-EC14142-usp, respectively (accessed on 29 April 2022).

## Supplementary Materials

The following supporting information can be downloaded at: https://www.mdpi.com/article/10.3390/antibiotics11111620/s1, Table S1: Assembly statistics generated through WGS of UPEC isolate EC14142 from Egypt.
