# Supplementary material for: Pathogenicity Islands in Uropathogenic Escherichia coli Clinical Isolate of the Globally Disseminated O25:H4-ST131 Pandemic Clonal Lineage: First Report from Egypt
Source: Antibiotics (Basel). 2022 Nov 13;11(11):1620. doi: 10.3390/antibiotics11111620 (PMC9686529; doi:10.3390/antibiotics11111620)
Supplement: Supplementary file 1 [file antibiotics-11-01620-s001.zip › antibiotics-2003284-supplementary.pdf]

Supplementary Table S1: Assembly statistics generated through WGS of UPEC isolate EC14142 from Egypt.

Depth of coverage	218x
Genome breadth (%)	99.2
Number of reads	5,167,285
Total length of sequences	5,936,425
Total number of contigs	296
N50 (bp)	85375
GC (%)	51.2
CDSs	4,728
5s, 16s, 23rrna	1, 1, 1
